# Large increase of vertebral osteomyelitis in France: a 2010–2019 cross-sectional study

**DOI:** 10.1017/S0950268821002181

**Published:** 2021-10-06

**Authors:** Yoann Conan, Emeline Laurent, Yannick Belin, Marion Lacasse, Aymeric Amelot, Denis Mulleman, Philippe Rosset, Louis Bernard, Leslie Grammatico-Guillon

**Affiliations:** 1 Department of Public Health, Unit of Clinical Epidemiology (EpiDcliC), Teaching Hospital of Tours, Tours, France; 2 Department of Infectious Diseases, Teaching Hospital of Tours, Tours, France; 3 Medical School, University of Tours, Tours, France; 4 Research Unit EA7505 (Education Ethique et Santé), University of Tours, Tours, France; 5 Department of Neurosurgery, Teaching Hospital of Tours, Tours, France; 6 Department of Rheumatology, Teaching Hospital of Tours, Tours, France; 7 Department of Orthopaedic Surgery, Teaching Hospital of Tours, Tours, France; 8 Reference Centre for Complex Bone and Joint Infections of Western France (CRIOGO), Tours, France

**Keywords:** Bone infections, device-associated infection, epidemiology, hospital discharge databases, vertebral osteomyelitis

## Abstract

Vertebral osteomyelitis (VO) represents 4–10% of bone and joint infections. In Western countries, its incidence seems to increase, simultaneously with an increasing number of comorbidities among an ageing population. This study aimed to assess the evolution of VO epidemiology in France over the 2010–2019 decade. A nationwide cross-sectional study was conducted using the French hospital discharge data collected through the French diagnosis-related groups ‘*Programme de Médicalisation des Systèmes d'Information*’. VOs were detected with a previously validated case definition using International Classification of Diseases 10 (ICD-10) codes, implemented with the French current procedural terminology codes. The study population included all patients hospitalised in France during the 2010–2019 decade, aged 15 years old and more. Patient and hospital stay characteristics and their evolutions were described. During the study period, 42 105 patients were hospitalised for VO in France involving 60 878 hospital stays. The mean VO incidence was 7.8/100 000 over the study period, increasing from 6.1/100 000 in 2010 to 11.3/100 000 in 2019. The mean age was 64.8 years old and the sex ratio was 1.56. There were 31 341 (74.4%) patients with at least one comorbidity and 3059 (7.3%) deceased during their hospital stay. Even if rare, device-associated VOs (4450 hospital stays, 7.3%) highly increased over the period. The reliability of the method, based upon an exhaustive database and a validated case definition, provided an effective tool to compare data over time in real-life conditions to regularly update the epidemiology of VO.

## Introduction

Vertebral osteomyelitis (VO), also named infectious spondylodiscitis, is the infection of an intervertebral disc and at least one adjacent vertebra. It is associated with considerable morbidity, disability and extra costs [1–5]. Haematogeneous dissemination represents the main infection process (60–80%) while direct and local inoculation following a spinal surgery, a lumbar puncture or an epidural injection is less frequent (15–40%) [1, 6]. Clinical presentation is often characterised by non-specific symptoms (back pain, back stiffness, fever), especially within the elderly with multiple comorbidities [1, 6]. Following a recent spinal surgery, local scarring signs are commonly observed [5]. VO is a serious infection requiring a hospital management, an antibiotic treatment and a potential surgical treatment [1, 5, 6].

Since several years, many studies suggested an increase in VO incidence in Western countries [3, 7–11]. This might be explained by the increasing number of comorbidities in those countries, especially among the ageing population, or by an improvement in the diagnosis of VO [10–12]. Indeed, an enhancement in imaging techniques and higher availability of magnetic resonance imaging (MRI) could have improved the diagnosis of VO [7, 10, 12]. The French infectious diseases society (SPILF) lastly updated its guidelines in 2007, potentially leading to better management of patients with suspected or confirmed VO in France [6]. It was followed by the American society (IDSA) in 2015 then by three European societies (EANM, ESNR and ESCMID) in 2019 [13, 14].

In the light of these evolutions, updating the epidemiology of VO in France appeared to be a necessity to better understand the patients with VO and the consequences regarding their hospital management. The study aimed to assess the evolution of VO incidence in France over the last decade and describe the patient outcomes using the French hospital discharge databases.

## Methods

### Study design

A nationwide cross-sectional study was conducted over the 2010–2019 period, using the French hospital discharge (HD) data available on the national secured website of the *Agence Technique de l'Information Hospitalière* (*ATIH*). VOs were detected with a previously validated algorithm developed from the French diagnosis-related groups (DRG) *Programme de Médicalisation des Systèmes d'Information* (*PMSI*), giving a 94% predictive positive value [2].

This national HD database is based on the mandatory notification of each hospital stay for all public or private hospitals. All hospitalisation information is stored in a coded summary using the International Classification of Diseases 10 (ICD-10) and the French current procedural terminology (CPT). All patients are assigned a unique identification number, allowing the same individual to be followed over time.

### Study population

Patients aged 15 years old (y.o.) or older meeting the criteria of the validated VO case definition (Supplementary data 1A) in their hospital resume over the 2010–2019 period were included. Indeed, we selected any HD with a principal or secondary diagnosis of VO appearing alone or in combination with either sepsis or a specific surgical procedure. International Classification of Diseases, Tenth Revision (ICD-10) codes included spondylodiscitis and infectious complications of surgical care (T codes). The surgical procedures according to the French Common Classification of Medical Acts (French CPT) included debridement, prosthesis removal, exchange or revision. Device-associated VO (DAVO) was defined as a VO with the presence and/or withdrawal of a spinal orthopaedic device (Supplementary data 1B).

We linked multiple hospitalisations to anonymised patient data, using a unique and encrypted patient number, in order to obtain the patient database. HD data were used to describe VO, whereas the patient database was used to describe the epidemiology of VO cases.

### Data analyses

Overall VO incidence was estimated by dividing the number of VO cases by the French population aged 15 y.o. or older over the decade, as estimated by the French *Institut National de la Statistique et des Etudes Economiques* (INSEE) and stratified by age and sex. VO incidence per year was estimated by dividing the number of VO cases during 1 year by the French population of the year.

Patient-related data included socio-demographics, comorbidities coded during the hospital stay (Supplementary data 2) and in-hospital mortality. An elderly patient was defined as a patient aged 65 y.o. or older.

Hospital stay-related data included the length of stay, healthcare sector (public or private), microbiological and antibiotic resistance codes and severity of the clinical presentation [severe sepsis, admission in intensive care unit (ICU)]. Hospital stays in reference centres for complex bone and joint infections (BJI) or presenting the specific ICD-10 code for complex BJI (Z 76 800 assigned after validation from a multidisciplinary team) were also described as previously studied [4]. Patient and hospital stay costs were described without adjustment.

Data were described according to their number and rate for qualitative variables and according to their mean, median, minimum and maximum for quantitative variables, as well as quartiles, fifth and 95th percentiles for hospital length of stay and costs. The analysis was first conducted for the overall study population, then specifically among DAVO hospital stays. No statistical test was performed, as all comparisons were likely to be statistically different regardless of clinical significance and the *PMSI* discharge database is exhaustive.

### Ethic statements

The study was performed in accordance with the French Reference Methodology MR-005 for retrospective studies using PMSI, elaborated by the French authority *Commission Nationale de l'Informatique et des Libertés* (*CNIL*). It was registered on the Health Data Hub, number F20210121134244.

## Results

**Fig. 1.**
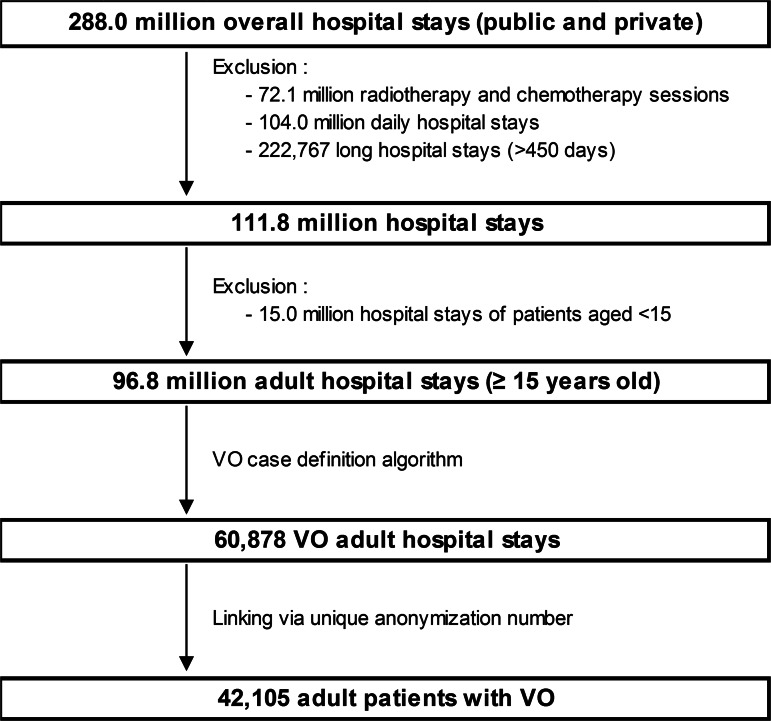
Flow chart of the study population (adult patients with vertebral osteomyelitis VO).

During the study period, 42 105 patients aged 15 y.o. or older were hospitalised for VO in France (Fig. 1), involving 60 878 hospital stays (mean 1.43 hospital stay per patient). The patients were mainly male (60.9%), mean age was 64.8. Among them, 3059 (7.3%) died during their VO stay. Nearly 10% had a DAVO (*N* = 3881) (Table 1).

**Table 1.** Characteristics of vertebral osteomyelitis (VO) patients

		Total
	*N*	%
Number of patients (*n*)	**42 105**	***100***
Age		
Mean	64.8	
[Minimum – Maximum]	[15–101]	
Sex-ratio M/F	1.56	
Male	25 644	*60*.*9*
Female	16 461	*39*.*1*
Device-associated VO	3881	*9.2*
Comorbidities (*n*)	31 341	*74.4*
0	10 764	*25*.*6*
1	10 122	*24*.*0*
2	8498	*20*.*2*
≥3	12 721	*30*.*2*
In-hospital mortality	3059	*7.3*
Hospital stays per patient		
Mean	1.43	
Median	1	
[Minimum – Maximum]	[1–24]	
Cost per patient		
Mean	15 393	
Median	11 005	
[Minimum – Maximum]	[18–598 591]	

### Incidence evolution and patient description

**Fig. 2.**
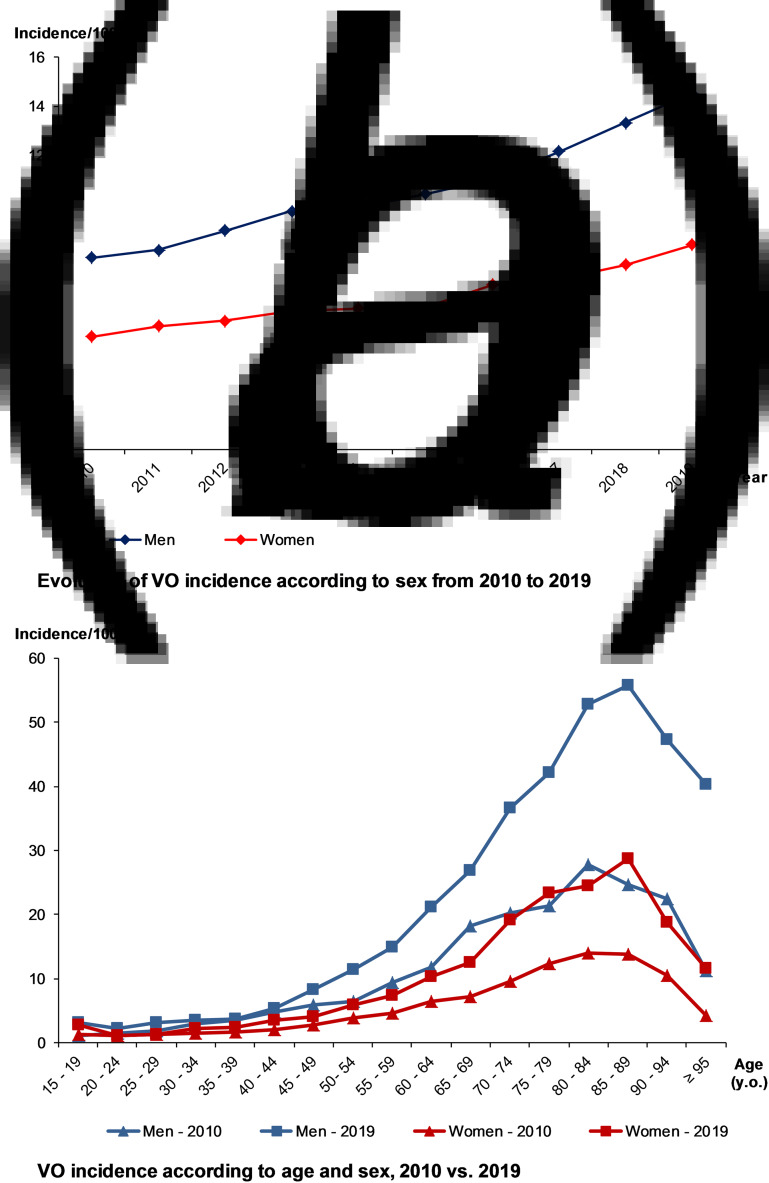
Evolution of the incidence of vertebral osteomyelitis (VO) over the decade, by age and sex.

Over 2010–2019, VO incidence was 7.8/100 000, higher in males than females (10.0/100 000 *vs.* 5.9/100 000). Over the decade, it nearly doubled, from 6.1/100 000 to 11.3/100 000 (+84%) (Fig. 2a) and was higher in the elderly (Fig. 2b). The maximum incidence was observed for people aged between 75 and 90 y.o., regardless of the gender or the year. When focusing on residency areas and administrative French regions, we also observed heterogeneity of incidence rate, after standardisation on age and sex. However, whatever the region, an increasing incidence rate was observed over the decade, comparing the two periods of 5 years (Supplementary data 3).

Three-quarters of the patients had one or more comorbidities, and 30.2% had three or more (Table 1). Cardiovascular diseases were the most common comorbidities (39.7%), followed by diabetes mellitus (19.8%). Kidney failures (15.4%), urinary tract infections (14.8%), cancers (13.7%) and obesity (10.2%) were also commonly identified.

Also, 2102 patients (3.5%) had severe sepsis and 1414 (2.3%) were hospitalised in ICU. Endocarditis was retrieved in 11.5% of the patients.

### Hospital stay characteristics and evolution

During the study period, there were 60 878 adult VO hospital stays, increasing each year and nearly doubling between 2010 and 2019, from 4382 in 2010 to 8487 in 2019 (+94%) (Table 2). Most of the hospital stays (87.8%) occurred in a public healthcare facility, 31.8% occurred in a reference centre for complex BJI and 28.5% in a surgical unit.

**Table 2.** Characteristics of the hospital stays for vertebral osteomyelitis (VO)

	Native VO		Device-associated VO		Total	
	*N*	%^a^	*N*	%^a^	*N*	
Hospital stays	56 428	*92.7*	4450	*7.3*	60 878	
2010	4175	*95.3*	207	*4.7*	4382	
2011	4504	*95.3*	224	*4.7*	4728	
2012	4762	*94.8*	260	*5.2*	5022	
2013	5123	*93.9*	332	*6.1*	5455	
2014	5265	*93.6*	363	*6.4*	5628	
2015	5320	*92.2*	452	*7.8*	5772	
2016	5973	*92.1*	512	*7.9*	6485	
2017	6500	*91.5*	601	*8.5*	7101	
2018	7076	*90.5*	742	*9.5*	7818	
2019	7730	*91.1*	757	*8.9*	8487	
	*N*	% ^b^	*N*	% ^c^	*N*	%^d^
Microbiological evidence	37 647	*66.7*	3269	*73.5*	40 916	*67.2*
Bacteria	37 107	*65.8*	3241	*72.8*	40 348	*66,3*
Polybacteraemia	6199	*11.0*	696	*15.6*	6895	*11,3*
*Staphylococci*	18 957	*33.6*	2447	*55.0*	21 404	*35*.*2*
*Gram-negative, bacilli*	9854	*17.5*	939	*21.1*	10 793	*17*.*7*
*Streptococci*	9025	*16.0*	454	*10.2*	9479	*15*.*6*
*Tuberculosis*	3936	*7.0*	11	*0.2*	3947	*6*.*5*
Resistance	6970	*12.4*	851	*19.1*	7821	*12,8*
Severe sepsis	1973	*3.5*	129	*2.9*	2102	*3.5*
Intensive care unit	1265	*2.2*	149	*3.3*	1414	*2.3*
Reference centre	17 273	*30.6*	2090	*47.0*	19 363	*31.8*
Code Z 76 800	869	*1.5*	404	*9.1*	1273	*2.1*
Surgical unit	13 697	*24.3*	3628	*81.5*	17 325	*28.5*
Public sector	49 897	*88.4*	3530	*79.3*	53 427	*87.8*

a % of all VO of the current year.

b % of native VO.

c % of device-associated VO.

d % of all VO.

A microorganism was coded in 40 916 hospital stays (67.2%). Gram-positive cocci were the most frequent bacteria: 21 404 Staphylococci (52.3% of hospital stays with microbiological codes) and 9479 Streptococci (23.2%). Gram-negative bacilli were found in 10 793 hospital stays (26.4%) and tuberculosis in 3947 (9.6%) (Table 2).

DAVOs remained rare (4450 hospital stays, 7.3%) (Table 2), but highly increased in both number and rate over the period. Most of them required hospitalisation in a surgical unit (81.5% *vs.* 28.5% of all VO hospital stays). As compared to native VOs, hospitalisation in a reference centre and hospitalisation in a private healthcare facility were more frequent among DAVOs. DAVOs were less often associated with severe sepsis than native VOs, but more often admitted to ICU. A microorganism was more often coded in DAVOs than in native VOs (73.5% of the DAVOs): *Staphylococci* were the most common followed by Gram-negative bacilli while Streptococci were less frequent. Polybacteraemia and antibioresistance were also more frequent (Table 2).

**Fig. 3.**
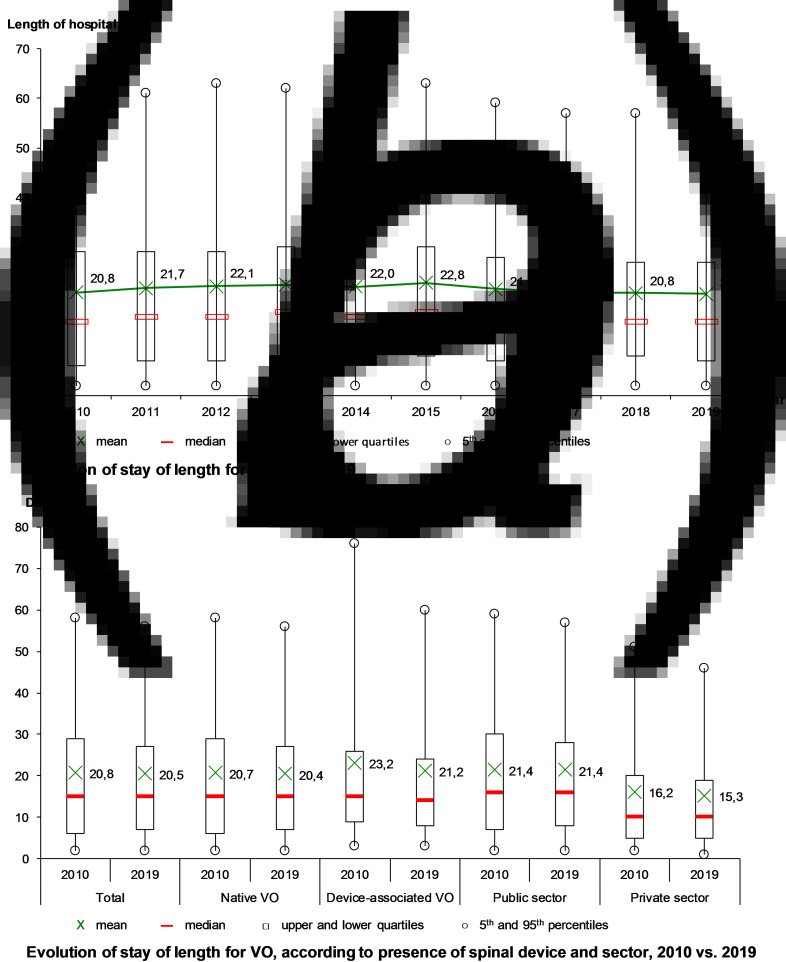
Evolution of hospital stays for vertebral osteomyelitis (VO) over the decade, France.

The mean length of stay was 21.4 days, stable from 20.8 days in 2010 to 20.5 days in 2019, with a maximum in 2015 (22.8 days) (Fig. 3a). Over the decade, the mean length of stay decreased for DAVO (from 23.2 to 21.2 days), especially for the longest stays (from 77 to 60 days in the 95th percentile) and in the private sector (from 16.2 to 15.3 days) (Fig. 3b).

The mean cost of VO hospital stay was 10 798 euros. It gradually increased from 9955 euros in 2011 to 10 950 in 2019, regardless of inflation. The mean cost for DAVO remained stable at around 13 000 euros per stay. However, it decreased in very expensive stays (from 31 619 to 27 495 euros in the 95th percentile). As compared with the public sector, these crude costs were about twice as low in the private sector, but slightly increased over the study period (from 5020 to 5462 euros).

## Discussion

VO incidence increased in France from 2010 to 2019, rising from 6.1/100 000 to 11.3/100 000, predominantly in elderly men with multiple comorbidities. This study highlighted the frailty of VO patients and the substantial increase of DAVO. Our study is one of the first to date to use a large real-life database with the completeness of the cases collection, allowing an accurate estimation of the incidence. A similar increase in VO incidence has been found in Western countries [3, 7, 9–11]. In another Japanese study using an administrative database over a 4-year period, an increasing trend was found with a VO incidence rising from 5.3/100 000 population per year in 2007 to 7.4/100 000 population per year in 2010 [11]. In a US epidemiological study over 15 years, the incidence of VO admission was 4.8 per 100 000, increasing from 8021 cases (2.9/100 000) in 1998 to 16 917 cases (5.4/100 000) in 2013 [9]. Moreover, in a population-based study, focused on 1995–2008 pyogenic VO in a Danish county, the overall incidence increased from 2.2 to 5.8 cases per 100 000 person-years [10]. The main hypotheses to explain this evolution could be the frailty of the ageing population and the improvement in VO diagnosis [7, 9–11]. Indeed, in France in 2007, the SPILF guidelines update for VO diagnosis and management probably led to better clinical awareness and understanding, which are key determinants to improve diagnosis and could be linked to an incidence increase [6]. The more systematic use of MRI for patients with compatible clinical signs, even with more subtle presentation as in elderly for whom back pain and stiffness are non-specific, may have taken part in this increase [7, 10, 12]. Indeed, a recent Spanish retrospective study suggested an increased diagnosis of VO with subtle clinical presentation, which was more frequently associated with the absence of microbiological evidence and a more prominent role of less virulent bacteria [8]. However, caution must be taken in interpreting these findings, especially in the elderly for whom it could be challenging to differentiate VO and erosive degenerative disc diseases, potentially leading to over-diagnose VO [6].

Our study showed that patients with VO were more likely to be male, aged and frail, as estimated in other Occidental studies [7, 9, 10]. Indeed in the Danish study, the elderly had the highest incidence compared to those aged ≤70 years (rate ratio for men 5.9 [95% confidence interval (CI) 4.2–8.5] and for women 3.5 (95% CI 2.3–5.3)) [10] as in the Japanese study where 58.9% of VO patients were men and the average age was 69.2 years [11]; whereas a majority of patients of the US epidemiological database study from 1998 and 2013 were male (51%) and younger than 59 years of age (49.5%) [9]. In Western countries, both life expectancy and mean age are increasing [15, 16]. However, VO incidence was also increasing in every age group, so the ageing of the population cannot explain these results by itself. Most of the patients had comorbidities, which increased from 2010 to 2019. These evolutions should be interpreted in the light of the coding process that became more comprehensive and efficient because of its professionalisation in France and a financial incentive. However, the main comorbidities we identified are already known as diseases with an increasing prevalence over the last decades like in previous international studies, including diabetes mellitus, cancer, intravenous drug use, alcoholism or cirrhosis [7, 9, 10, 17]. Eventually, the differences we observed among French regions suggest that environmental and demographic factors may have taken part in these evolutions. Though, our study design could not allow us to identify these factors.

A substantial increase in the incidence of DAVOs has been retrieved. Even if starting from an extremely low number in 2010, the DAVO rate nearly doubled over the decade. A parallel increase in spinal surgeries is observed in many Western countries, in order to face the rising demand for functional surgery especially concerning degenerative and non-traumatic spinal diseases in the elderly [5, 18–20].

The microbiologically confirmed VO rate remained stable as compared to the previous studies using *PMSI* [2, 4], even with the SPILF guidelines recommending a more active seeking for microbiological diagnosis. Other European studies found the same stability of microbiologically confirmed VOs over recent years [10, 21].

The 2007 French guidelines recommended to reduce the duration of immobilisation in non-complicated VOs [6]. Also, a recent clinical trial suggested that the duration of antibiotic treatments and supine position could be shortened [22]. Thus, a faster discharge home and a decrease in the length of stay were expected. Yet, the mean length of stay decreased by 4 days compared to the 2002–2003 study but it remained quite stable over the decade [2]. Still, we observed a decrease in long hospital stays which were more likely to concern severe or complicated VO cases, suggesting better management of these cases.

This study has some limitations, notably, the controversial issue concerning the reliability of the coding system, as data are coded by different healthcare professionals [2, 3, 23]. VO may be perceived more relevant by physicians, and so priority coded in the discharge resume, than others such as microbiological evidence. However, variation of coding practice was assessed amongst medical practitioners [24], providing strong predictive values. Recent studies used robust medical information systems in association with surveillance network data and obtained good results with a sensitivity/specificity and predictive positive value of 95%, 99% and 84%, respectively [3, 25]. In our study, the algorithm for defining a VO case was built by a multidisciplinary team using different combinations of codes of interest for spine-related infections hospital stays. This kind of data reuse for epidemiological purposes has shown robust performance for the detection and surveillance of numerous diseases [3, 9, 10, 23]. Another limit of our study was that we could not identify infection sources and processes (haematogeneous or local) through the PMSI data. We also found limits in using a medico-administrative database, especially concerning the identification of factors explaining these evolutions. For instance, the deprivation status of the patients could not be retrieved in the study design, whereas it could potentially enhance the frailty, a key determinant of VO occurrence.

Despite some limits, HDD-based surveillance could be promoted as a cost-effective method for routine surveillance, especially in infectious diseases. The validated algorithm represented one of the main strengths of the study by providing an exhaustive and real-life review of VO cases to compare VO incidence over time. Using the same method over the decade, an update of the VO epidemiology was performed [12]. As compared to the 2002–2003 results [2], VO incidence was 2.8/100 000, 6.1 in 2010 and 11.3 in 2019 in the current study. Even though ICD-10 and procedures codes for DAVOs were not included in the initial algorithm, the 2002–2003 VO incidence was probably not underestimated, as these DAVOs were most probably coded along with overall VOs. Cost-benefit analysis and studies combining multiple hospital databases are furthermore warranted and an automated surveillance system could be thought, especially to improve an analysis of the potential explicative factors that the HDD could not identify.

To conclude, our study outlined the high increase in VO and DAVO incidence in France over the 2010–2019 decade, similarly to other Western countries. The reliability of the method, based upon an exhaustive database and a validated case definition, provided an effective tool to compare data over time in real-life conditions to regularly update the epidemiology of VO. It also highlighted the potential need for an update in the current French guidelines, especially concerning epidemiological and microbiological diagnosis trends.

## Data Availability

Data are available on the national secured website of the ‘Agence Technique de l'Information Hospitalière’ (ATIH : https://www.atih.sante.fr/) for researchers who meet the criteria for access to confidential data.

## Supplementary material

For supplementary material accompanying this paper visit http://dx.doi.org/10.1017/S0950268821002181. click here to view supplementary material
