# Supplementary material for: Large increase of vertebral osteomyelitis in France: a 2010–2019 cross-sectional study
Source: Epidemiol Infect. 2021 Oct 6;149:e227. doi: 10.1017/S0950268821002181 (PMC8569834; doi:10.1017/S0950268821002181)
Supplement: Supplementary file 1 [file hygsup.zip › S0950268821002181sup001.docx]

**Supplementary data S1**

**A. Vertebral osteomyelitis – *PMSI* case definition**

| **At least one ICD-10 code among:** | | |
| --- | --- | --- |
|  | M46.2 | Osteomyelitis of vertebra |
|  | M46.3 | Infection of intervertebral disc (pyogenic) |
|  | M46.5 | Other infective spondylopathies |
|  | M49.0 | Tuberculosis of spine |
|  | M49.1 | Brucella spondylitis |
|  | M49.2 | Enterobacterial spondylitis |
|  | M49.3 | Spondylopathy in other infectious and parasitic diseases classified elsewhere |

**OR**

| **At least one procedure code among:** (French current procedural terminology *CCAM*: in French) | | |
| --- | --- | --- |
|  | AFJA002 | Parage d'une épidurite et/ou d'une spondylodiscite sans reconstruction vertébrale, par abord direct |
|  | AFJA005 | Parage d'une épidurite et/ou d'une spondylodiscite avec reconstruction vertébrale par greffe et/ou ostéosynthèse, par abord direct |
|  | LHPA004 | Mise à plat d'une lésion vertébrale infectieuse ou ossifluente, par abord postérieur |

**OR**

| **At least one ICD-10 code among:** | | | |
| --- | --- | --- | --- |
|  | T84.6 | | Infection and inflammatory reaction due to internal fixation device |
|  | T84.7 | | Infection and inflammatory reaction due to other internal orthopaedic prosthetic devices, implants and grafts |
|  | | **AND** | |
| **At least one procedure code among:** (French current procedural terminology *CCAM*: in French) | | | |
|  | LDGA001 | | Ablation de matériel d'ostéosynthèse de l'atlas et/ou de l'axis, par cervicotomie |
|  | LDGA002 | | Ablation de matériel d'ostéosynthèse de la colonne vertébrale, par cervicotomie antérieure ou antérolatérale |
|  | LEGA001 | | Ablation de matériel d'ostéosynthèse de la colonne vertébrale, par thoraco-phréno-laparotomie |
|  | LEGA002 | | Ablation de matériel d'ostéosynthèse de la colonne vertébrale, par thoracotomie |
|  | LFGA001 | | Ablation de matériel d'ostéosynthèse de la colonne vertébrale, par laparotomie ou par lombotomie |
|  | LHGA004 | | Ablation de matériel d'ostéosynthèse de la colonne vertébrale sur 10 vertèbres ou plus, par abord postérieur |
|  | LHGA006 | | Ablation de matériel d'ostéosynthèse de la colonne vertébrale sur 6 à 9 vertèbres, par abord postérieur |
|  | LHGA007 | | Ablation de matériel d'ostéosynthèse de la colonne vertébrale sur 2 à 5 vertèbres, par abord postérieur |

**B. Device-associated vertebral osteomyelitis – *PMSI* case definition**

| **At least one ICD-10 code among:** | | |
| --- | --- | --- |
|  | T84.6 | Infection and inflammatory reaction due to internal fixation device |
|  | T84.7 | Infection and inflammatory reaction due to other internal orthopaedic prosthetic devices, implants and grafts |

**AND**

| **At least one procedure code among:** (French current procedural terminology *CCAM*: in French) | | | |
| --- | --- | --- | --- |
|  | LDGA001 | | Ablation de matériel d'ostéosynthèse de l'atlas et/ou de l'axis, par cervicotomie |
|  | LDGA002 | | Ablation de matériel d'ostéosynthèse de la colonne vertébrale, par cervicotomie antérieure ou antérolatérale |
|  | LEGA001 | | Ablation de matériel d'ostéosynthèse de la colonne vertébrale, par thoraco-phréno-laparotomie |
|  | LEGA002 | | Ablation de matériel d'ostéosynthèse de la colonne vertébrale, par thoracotomie |
|  | LFGA001 | | Ablation de matériel d'ostéosynthèse de la colonne vertébrale, par laparotomie ou par lombotomie |
|  | LHGA004 | | Ablation de matériel d'ostéosynthèse de la colonne vertébrale sur 10 vertèbres ou plus, par abord postérieur |
|  | LHGA006 | | Ablation de matériel d'ostéosynthèse de la colonne vertébrale sur 6 à 9 vertèbres, par abord postérieur |
|  | LHGA007 | | Ablation de matériel d'ostéosynthèse de la colonne vertébrale sur 2 à 5 vertèbres, par abord postérieur |
|  | | **OR** | |
| **At least one ICD-10 code among:** | | | |
|  | M46.2 | | Osteomyelitis of vertebra |
|  | M46.3 | | Infection of intervertebral disc (pyogenic) |
|  | M46.5 | | Other infective spondylopathies |
|  | M49.0 | | Tuberculosis of spine |
|  | M49.1 | | Brucella spondylitis |
|  | M49.2 | | Enterobacterial spondylitis |
|  | M49.3 | | Spondylopathy in other infectious and parasitic diseases classified elsewhere |
