# Supplementary material for: Large increase of vertebral osteomyelitis in France: a 2010–2019 cross-sectional study
Source: Epidemiol Infect. 2021 Oct 6;149:e227. doi: 10.1017/S0950268821002181 (PMC8569834; doi:10.1017/S0950268821002181)
Supplement: Supplementary file 1 [file hygsup.zip › S0950268821002181sup002.docx]

**Supplementary data S2**

**Comorbidities – ICD-10 codes used**

| **ICD-10 code** | | **Diagnosis** |
| --- | --- | --- |
|  | | **Diabetes** |
|  | E10 – E14 | Diabetes mellitus |
|  | G59.0 | Diabetic mononeuropathy |
|  | G63.2 | Diabetic polyneuropathy |
|  | M14.2 | Diabetic arthropathy |
|  | N08.3 | Glomerular disorders in diabetes mellitus |
|  |  | **Chronic wound** |
|  | I83.0 | Varicose veins of lower extremities with ulcer |
|  | I83.2 | Varicose veins of lower extremities with both ulcer and inflammation |
|  | L89 | Decubitus ulcer and pressure area |
|  | L97 | Ulcer of lower limb, not elsewhere classified |
|  | L98.4 | Chronic ulcer of skin, not elsewhere classified |
|  |  | **Peripheral vascular disorders** |
|  | I70 | Atherosclerosis |
|  | I73 | Other peripheral vascular diseases |
|  | I74.2 | Embolism and thrombosis of arteries of upper extremities |
|  | I74.3 | Embolism and thrombosis of arteries of lower extremities |
|  | I74.4 | Embolism and thrombosis of arteries of extremities, unspecified |
|  | I74.5 | Embolism and thrombosis of iliac artery |
|  | I74.8 | Embolism and thrombosis of other arteries |
|  | I74.9 | Embolism and thrombosis of unspecified artery |
|  | I77.1 | Stricture of artery |
|  | I77.5 | Necrosis of artery |
|  | I77.6 | Arteritis, unspecified |
|  | I77.8 | Other specified disorders of arteries and arterioles |
|  | I77.9 | Disorder of arteries and arterioles, unspecified |
|  | I79.2 | Peripheral angiopathy in diseases classified elsewhere |
|  | I79.8 | Other disorders of arteries, arterioles and capillaries in diseases classified elsewhere |
|  | I80 | Phlebitis and thrombophlebitis |
|  | I82.8 | Embolism and thrombosis of other specified veins |
|  | I82.9 | Embolism and thrombosis of unspecified vein |
|  | I83.1 | Varicose veins of lower extremities with inflammation |
|  | I83.9 | Varicose veins of lower extremities without ulcer or inflammation |
|  | I87 | Other disorders of veins |
|  |  | **Alcohol use** |
|  | E24.4 | Alcohol-induced pseudo-Cushing syndrome |
|  | F10 | Mental and behavioural disorders due to use of alcohol |
|  | G31.2 | Degeneration of nervous system due to alcohol |
|  | G62.1 | Alcoholic polyneuropathy |
|  | G72.1 | Alcoholic myopathy |
|  | I42.6 | Alcoholic cardiomyopathy |
|  | K29.2 | Alcoholic gastritis |
|  | K70 | Alcoholic liver disease |
|  | K85.2 | Alcohol-induced acute pancreatitis |
|  | K86.0 | Alcohol-induced chronic pancreatitis |
|  | Z50.2 | Alcohol rehabilitation |
|  | Z71.4 | Alcohol abuse counselling and surveillance |
|  | Z72.1 | Alcohol use |
|  |  | **Smoking** |
|  | F17 | Mental and behavioural disorders due to use of tobacco |
|  | T65.2 | Toxic effects of tobacco and nicotine |
|  | Z71.6 | Tobacco abuse counselling |
|  | Z72.0 | Tobacco use |
|  |  | **Drug abuse** |
|  | F11 | Mental and behavioural disorders due to use of opioids |
|  | F12 | Mental and behavioural disorders due to use of cannabinoids |
|  | F13 | Mental and behavioural disorders due to use of sedatives or hypnotics |
|  | F14 | Mental and behavioural disorders due to use of cocaine |
|  | F15 | Mental and behavioural disorders due to use of other stimulants, including caffeine |
|  | F16 | Mental and behavioural disorders due to use of hallucinogens |
|  | F19 | Mental and behavioural disorders due to multiple drug use and use of other psychoactive substances |
|  | Z50.3 | Drug rehabilitation |
|  | Z71.5 | Drug abuse counselling and surveillance |
|  | Z72.2 | Drug use |
|  |  | **Cancer** |
|  | C00 – C97 | Malignant neoplasms |
|  | D63.0 | Anaemia in neoplastic disease |
|  | E34.0 | Carcinoid syndrome |
|  | G13.1 | Other systemic atrophy primarily affecting central nervous system in neoplastic disease |
|  | G53.3 | Multiple cranial nerve palsies in neoplastic disease |
|  | G55.0 | Nerve root and plexus compressions in neoplastic disease |
|  | G73.2 | Other myasthenic syndromes in neoplastic disease |
|  | G94.1 | Hydrocephalus in neoplastic disease |
|  | M36.0 | Dermato(poly)myositis in neoplastic disease |
|  | M36.1 | Arthropathy in neoplastic disease |
|  | M90.6 | Osteitis deformans in neoplastic disease |
|  | M90.7 | Fracture of bone in neoplastic disease |
|  |  | **HIV infection** |
|  | B20 – B24 | Human immunodeficiency virus [HIV] disease |
|  | F02.4 | Dementia in human immunodeficiency virus [HIV] disease |
|  | Z21 | Asymptomatic human immunodeficiency virus [HIV] infection status |
|  |  | **Liver disease** |
|  | B18 | Chronic viral hepatitis |
|  | B19 | Unspecified viral hepatitis |
|  | E83.1 | Disorders of iron metabolism |
|  | I85 | Oesophageal varices |
|  | I98.2 | Oesophageal varices without bleeding in diseases classified elsewhere |
|  | K70 | Alcoholic liver disease |
|  | K71 | Toxic liver disease |
|  | K72 | Hepatic failure, not elsewhere classified |
|  | K73 | Chronic hepatitis, not elsewhere classified |
|  | K74 | Fibrosis and cirrhosis of liver |
|  | K76 | Other diseases of liver |
|  | Q44.6 | Cystic disease of liver |
|  | Q44.7 | Other congenital malformations of liver |
|  |  | **Organ transplant** |
|  | N16.5 | Renal tubulo-interstitial disorders in transplant rejection |
|  | T86 | Failure and rejection of transplanted organs and tissues |
|  | Z94.0 | Kidney transplant status |
|  | Z94.1 | Heart transplant status |
|  | Z94.2 | Lung transplant status |
|  | Z94.3 | Heart and lungs transplant status |
|  | Z94.4 | Liver transplant status |
|  | Z94.6 | Bone transplant status |
|  | Z94.8 | Other transplanted organ and tissue status |
|  |  | **Cardiovascular diseases** |
|  | I10 | Essential (primary) hypertension |
|  | I11 | Hypertensive heart disease |
|  | I12.9 | Hypertensive renal disease without renal failure |
|  | I13 | Hypertensive heart and renal disease |
|  | I15 | Secondary hypertension |
|  | I20 | Angina pectoris |
|  | I25 | Chronic ischaemic heart disease |
|  | I27 | Other pulmonary heart diseases |
|  | I42 | Cardiomyopathy |
|  | I43 | Cardiomyopathy in diseases classified elsewhere |
|  | I50 | Heart failure |
|  | I51.4 | Myocarditis, unspecified |
|  | I51.5 | Myocardial degeneration |
|  | I51.6 | Cardiovascular disease, unspecified |
|  | I51.8 | Other ill-defined heart diseases |
|  | I51.9 | Heart disease, unspecified |
|  | I52 | Other heart disorders in diseases classified elsewhere |
|  |  | **Presence of heart device** |
|  | T82.6 | Infection and inflammatory reaction due to cardiac valve prosthesis |
|  | T82.7 | Infection and inflammatory reaction due to other cardiac and vascular devices, implants and grafts |
|  | T82.8 | Other specified complications of cardiac and vascular prosthetic devices, implants and grafts |
|  | T82.9 | Unspecified complication of cardiac and vascular prosthetic device, implant and graft |
|  | Z95.0 | Presence of electronic cardiac devices |
|  | Z95.2 | Presence of prosthetic heart valve |
|  | Z95.3 | Presence of xenogenic heart valve |
|  | Z95.4 | Presence of other heart-valve replacement |
|  | Z95.8 | Presence of other cardiac and vascular implants and grafts |
|  | Z95.9 | Presence of cardiac and vascular implant and graft, unspecified |
|  |  | **Urinary tract infection or inflammation** |
|  | N10 | Acute tubulo-interstitial nephritis |
|  | N11 | Chronic tubulo-interstitial nephritis |
|  | N12 | Tubulo-interstitial nephritis, not specified as acute or chronic |
|  | N30.0 | Acute cystitis |
|  | N39.0 | Urinary tract infection, site not specified |
|  | N41 | Inflammatory diseases of prostate |
|  | T83.5 | Infection and inflammatory reaction due to prosthetic device, implant and graft in urinary system |
|  | T83.6 | Infection and inflammatory reaction due to prosthetic device, implant and graft in genital tract |
|  | T83.8 | Other complications of genitourinary prosthetic devices, implants and grafts |
|  | T83.9 | Unspecified complication of genitourinary prosthetic device, implant and graft |
|  | Z96.0 | Presence of urogenital implants |
|  |  | **Connective tissue disease** |
|  | G73.7 | Myopathy in other diseases classified elsewhere |
|  | M05 | Seropositive rheumatoid arthritis |
|  | M06 | Other rheumatoid arthritis |
|  | M08 | Juvenile arthritis |
|  | M31.5 | Giant cell arteritis with polymyalgia rheumatic |
|  | M31.6 | Other giant cell arteritis |
|  | M32 | Systemic lupus erythematosus |
|  | M33 | Dermatopolymyositis |
|  | M34 | Systemic sclerosis |
|  | M35 | Other systemic involvement of connective tissue |
|  |  | **Central paralysis** |
|  | G04.1 | Tropical spastic paraplegia |
|  | G11.4 | Hereditary spastic paraplegia |
|  | G80 – G83 | Cerebral palsy and other paralytic syndromes |
|  |  | **Obesity** |
|  | E66 | Obesity |
|  | T85.50 | Mechanical complication of perigastric devices and implants used in bariatric surgery |
|  |  | **Renal failure** |
|  | N17 – N19 | Renal failure |
|  |  | **Endocarditis** |
|  | B37.6 | Candidal endocarditis |
|  | I33 | Acute and subacute endocarditis |
|  | I38 | Endocarditis, valve unspecified |
|  | I39 | Endocarditis and heart valve disorders in diseases classified elsewhere |
