# Supplementary material for: Large increase of vertebral osteomyelitis in France: a 2010–2019 cross-sectional study
Source: Epidemiol Infect. 2021 Oct 6;149:e227. doi: 10.1017/S0950268821002181 (PMC8569834; doi:10.1017/S0950268821002181)
Supplement: Supplementary file 1 [file hygsup.zip › S0950268821002181sup003.docx]

**Supplementary data S3**

**
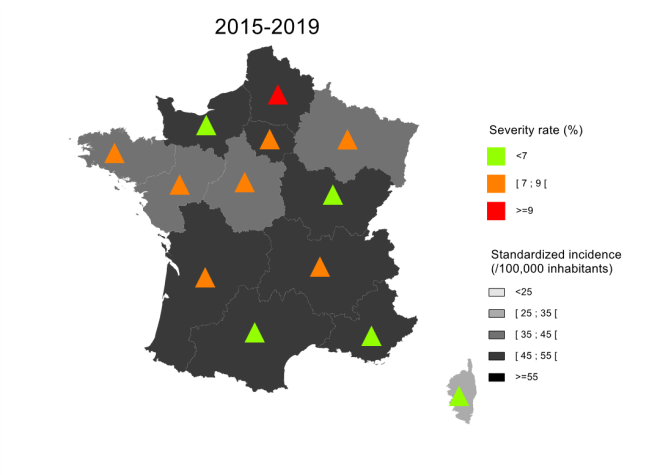

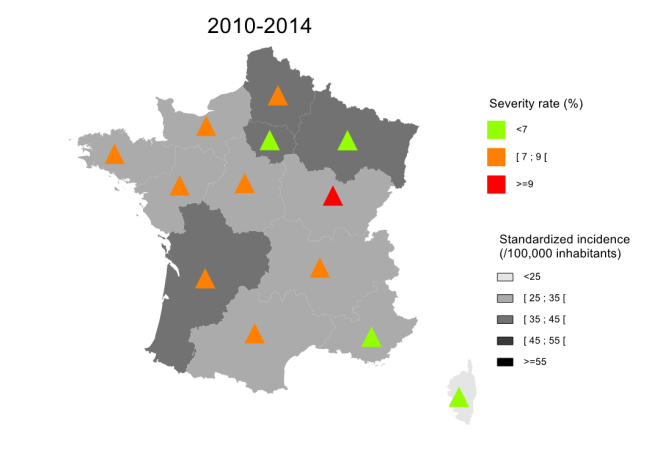
**

**S3 - Evolution of 5-year incidence standardized by age and sex and evolution of severity profile* by regions, over two 5-year periods, France**

**severity defined by coding severe sepsis and/or admission in intensive care unit or step unit*
